# P2X7 receptor contributes to long-term neuroinflammation and cognitive impairment in sepsis-surviving mice

**DOI:** 10.3389/fphar.2023.1179723

**Published:** 2023-04-21

**Authors:** Vinícius Santos Alves, Joyce Pereira da Silva, Fabiana Cristina Rodrigues, Suzana Maria Bernardino Araújo, André Luiz Gouvêa, Raíssa Leite-Aguiar, Stephanie Alexia Cristina Silva Santos, Milla Souza Pessoa da Silva, Fernanda Silva Ferreira, Eduardo Peil Marques, Beatriz Amanda Barbosa Rangel dos Passos, Tatiana Maron-Gutierrez, Eleonora Kurtenbach, Robson da Costa, Cláudia Pinto Figueiredo, Angela T. S. Wyse, Robson Coutinho-Silva, Luiz Eduardo Baggio Savio

**Affiliations:** ^1^ Instituto de Biofísica Carlos Chagas Filho, Universidade Federal do Rio de Janeiro, Rio de Janeiro, Brazil; ^2^ Faculdade de Farmácia, Universidade Federal do Rio de Janeiro, Rio de Janeiro, Brazil; ^3^ Laboratório de Neuroproteção e Doenças Metabólicas, Departamento de Bioquímica, Instituto de Ciências Básicas da Saúde (ICBS), Universidade Federal do Rio Grande do Sul, Porto Alegre, RS, Brazil; ^4^ Laboratório de Imunofarmacologia, Instituto Oswaldo Cruz, Fiocruz, Rio de Janeiro, Brazil

**Keywords:** sepsis-associated encephalopathy, P2X7 receptor, cognitive impairment, neuroinflammation, Brilliant Blue G, acetylcholinesterase

## Abstract

**Introduction:** Sepsis is defined as a multifactorial debilitating condition with high risks of death. The intense inflammatory response causes deleterious effects on the brain, a condition called sepsis-associated encephalopathy. Neuroinflammation or pathogen recognition are able to stress cells, resulting in ATP (Adenosine Triphosphate) release and P2X7 receptor activation, which is abundantly expressed in the brain. The P2X7 receptor contributes to chronic neurodegenerative and neuroinflammatory diseases; however, its function in long-term neurological impairment caused by sepsis remains unclear. Therefore, we sought to evaluate the effects of P2X7 receptor activation in neuroinflammatory and behavioral changes in sepsis-surviving mice.

**Methods:** Sepsis was induced in wild-type (WT), P2X7^−/−^, and BBG (Brilliant Blue G)-treated mice by cecal ligation and perforation (CLP). On the thirteenth day after the surgery, the cognitive function of mice was assessed using the novel recognition object and Water T-maze tests. Acetylcholinesterase (AChE) activity, microglial and astrocytic activation markers, and cytokine production were also evaluated.

**Results:** Initially, we observed that both WT and P2X7^−/−^ sepsis-surviving mice showed memory impairment 13 days after surgery, once they did not differentiate between novel and familiar objects. Both groups of animals presented increased AChE activity in the hippocampus and cerebral cortex. However, the absence of P2X7 prevented partly this increase in the cerebral cortex. Likewise, P2X7 absence decreased ionized calcium-binding protein 1 (Iba^−1^) and glial fibrillary acidic protein (GFAP) upregulation in the cerebral cortex of sepsis-surviving animals. There was an increase in GFAP protein levels in the cerebral cortex but not in the hippocampus of both WT and P2X7^−/−^ sepsis-surviving animals. Pharmacological inhibition or genetic deletion of P2X7 receptor attenuated the production of Interleukin-1β (IL-1β), Tumor necrosis factor-α (TNF-α), and Interleukin-10 (IL-10).

**Conclusion:** The modulation of the P2X7 receptor in sepsis-surviving animals may reduce neuroinflammation and prevent cognitive impairment due to sepsis-associated encephalopathy, being considered an important therapeutic target.

## 1 Introduction

Sepsis is a multifactorial syndrome characterized by an exacerbated inflammatory host immune response against infection, which can be lethal by inducing organ dysfunction and failure (Singer et al., 2016). Recently, this condition has been recognized as a priority global health problem due to its high incidence in developing countries (Reinhart et al., 2017; Rudd et al., 2020). Sepsis-associated encephalopathy (SAE) is a condition that patients can develop during the acute phase of sepsis. It is characterized by a diffuse cerebral dysfunction with symptoms ranging from confusion and delirium to coma (Prescott and Angus, 2018; Chung et al., 2020). Sepsis-surviving patients usually present long-term neurological sequelae after recovery, such as cognitive impairment and psychiatric disorders (e.i., depression and anxiety) (Iwashyna et al., 2010; Nikayin et al., 2016; Rabiee et al., 2016; Cavaillon et al., 2020).

The neuroinflammatory process can induce cell damage, promoting the release of damage-associated molecular patterns (DAMPs), such as ATP (Cauwels et al., 2014; Idzko et al., 2014). Extracellular ATP (eATP) acts as a danger signal molecule activating purinergic signaling via P2 receptors (Savio et al., 2018; Di Virgilio et al., 2020). The P2 receptors are divided into metabotropic receptors P2Y (P2Y_1_, P2Y_2_, P2Y_4_, P2Y_6_, and e P2Y_11-14_) and ionotropic receptors P2X (P2X1-7) (Fredholm et al., 2011; Illes et al., 2020). The P2X7 receptor has been described as the most relevant purinergic receptor in inflammatory processes (Di Virgilio et al., 2017). Evidence supports the involvement of P2X7 receptor in sepsis pathophysiology by contributing to the development of an excessive inflammatory response (Leite-Aguiar et al., 2021). However, P2X7 and P2X4 receptors might also be relevant for bacterial killing in sepsis (Csóka et al., 2018; Savio et al., 2018; Leite-Aguiar et al., 2021). Interestingly, P2X7 receptor expression increases on the surface of monocytes from septic patients (Martínez-García et al., 2019), and P2X7 receptor gain-of-function single nucleotide polymorphisms increased susceptibility to developing severe sepsis (Geistlinger et al., 2012).

Considering the importance of ATP-P2X7 receptor signaling in sepsis immune response, the relevance of this receptor has also been explored in SAE. The P2X7 receptor activation is involved in chemokine (C-X-C motif) ligand 1 (CXCL1) recruitment and brain endothelial activation, which promotes blood-brain barrier (BBB) leakage and microglial cell activation, causing neuroinflammation in the acute phase of sepsis (24 h) (Wang et al., 2015). In addition, the P2X7 receptor genetic deletion or pharmacological inhibition decreased pro-inflammatory cytokines IL-1β and IL-6 in the brain of septic mice, which suggests that this receptor may contribute to neuroinflammation and SAE development and severity (Savio et al., 2017). Even though evidence suggests the relevance of P2X7 receptor in acute sepsis-induced neurological symptoms, no studies have evaluated the long-term consequences of P2X7 activation in sepsis survivors. In this study, we sought to investigate the role of the P2X7 receptor in long-term brain alterations and cognitive impairment after a sepsis episode. We found that P2X7 receptor contributes to brain dysfunction in sepsis-surviving mice once the pharmacological blockade or genetic ablation of this receptor improved cognitive impairment and decrease glial markers and cytokine levels in the brain, which suggests the P2X7 receptors as an interesting therapeutic target for long-term neurological disturbances detected in sepsis survivors.

## 2 Materials and methods

### 2.1 Animals

Adult males aged 8–10 weeks, weighing between 25 and 30 g WT (wild-type) and P2X7 receptor knockout (P2X7^−/−^) C57BL/6 mice (originally from Jackson Laboratory, United States) were used. Animals were housed in groups of five per cage and with access to food and water *ad libitum* on a 12-h light/dark cycle in a room at a temperature of 22°C ± 1°C and controlled humidity. The procedures were performed following the guidelines of the Brazilian College of Animal Experimentation (COBEA) and were approved by The Commission for Ethical Use of Research Animals (CEUA) from the Federal University of Rio de Janeiro (UFRJ) (Protocol number: 073-19).

### 2.2 Sepsis induction by cecal ligation and puncture (CLP)

Polymicrobial sepsis induction was performed using the cecal ligation and puncture model (CLP) as previously described (Baker et al., 1983; Rittirsch et al., 2009). Briefly, mice were anesthetized with ketamine (80 mg/kg) and xylazine (20 mg/kg) given intraperitoneally (i.p.). Mice were shaved in the abdominal region and under aseptic condition; a laparotomy with a 1 cm incision was made on the abdomen. After the cecum exposure, it was ligated below the ileocecal region with a 4.0 mm silk thread. A double puncture was made in the cecum using a sterile 21-gauge needle, and a small amount of fecal content was gently squeezed out to induce peritonitis. The cecum was returned to the peritoneal cavity, and the abdominal wall was closed by suture. Sham animals (control group) were submitted to an identical surgical procedure without cecal ligation and perforation. After surgery, animals received 1 ml of 0.9% isotonic NaCl sterile solution subcutaneously to compensate for the fluids lost during the procedure. The brain and blood samples were collected 13 days after CLP induction.

### 2.3 Pharmacological treatments

All animals subjected to surgery were treated intraperitoneally (i.p) with the antibiotic Ertapenem (Invanz^®^) (75 mg/kg) 6, 24, 48, and 72 h after the surgical procedure. For the pharmacological blockade of the P2X7 receptor, mice were injected intraperitoneally with the P2X7 receptor-selective antagonist Brilliant Blue G (45 mg/kg) (from Sigma-Aldrich, MO, United States) or with vehicle control (Phosphate buffered saline (PBS) and dimethyl sulfoxide (DMSO 0.2%) 6 h, 3, 6, 9, and 12 days after sepsis induction (Savio et al., 2017). The animals were divided randomly into the following groups: WT Sham, P2X7^−/−^ Sham, WT CLP, P2X7^−/−^ CLP, WT Sham + Vehicle, WT Sham + BBG, WT CLP + Vehicle, and WT CLP + BBG.

### 2.4 Behavioral tests

Before all procedures, animals were habituated for 1 h with controlled temperature and light in the test room. Animals were evaluated 13 days after the CLP procedure. Additionally, experiments using animals that were not submitted to surgical procedures were performed to evaluate the basal cognitive ability of P2X7 receptor knockout mice.

#### 2.4.1 Novel object recognition test (NORT)

The novel object recognition test was performed in the arena measuring 30 × 30 × 45 cm to assess declarative memory. Test objects were fixed in the box floor using tape to avoid displacement caused by animals (De Sousa et al., 2021). None of the objects used in the test evoked innate preference. Animals were habituated and subjected to training and test sessions, which consisted of a 5-min long session each. The training session was performed by placing the animal in the center of the arena in the presence of two identical objects. The time that each animal spent exploring the objects was recorded. The exploratory behavior was considered when an animal sniffs and touches the object. After the training session, the objects were cleaned with 70% ethanol to eliminate olfactory cues. One hour after training, animals were placed in the same arena for a 5-min test session, and a new object replaced one of the objects used before. The time spent exploring familiar and novel objects was analyzed. Results were expressed as a recognition index and calculated by subtracting the time exploring the familiar object from the time exploring the novel object, divided by the total exploration time. Animals that learned the task presented recognition index >0.

#### 2.4.2 Water T-Maze test (WTMT)

The Water T-maze test was performed to assess working spatial memory. The apparatus consisted of a T-format arena with one main alley (60 cm length × 19 cm width) connected to two side arms (45 cm length × 19 cm width) (left and right). The apparatus was filled with water (23°C ± 1°C) and mice were placed at the beginning of the main alley and allowed to swim during 60 s for training. The side arm which mice entered was noted. For the main trials, a platform (11 cm height and 20 cm width), submerged 2 cm below the surface of water, was placed at the end of the opposite arm that mice preferred. The main trials consisted of five sessions of 60 s each, where mice were allowed to find the platform location. Once the platform was found, they remained on it for 20 s. If mice could not find the platform, they were gently guided to the location and remained for the same time indicated previously. The time the mice spent to find the platform was measured during each trial. An indication that mice learned the task was when they reached the platform in less time after each trial.

### 2.5 RNA extraction and real-time quantitative PCR (RT-qPCR)

Brain tissues were collected, and the cerebral cortex and hippocampus were isolated. TRIzol^®^ reagent (Thermo Fisher Scientific, Somerset, NJ, United States) was used for total RNA extraction from cerebral cortex structure according to the manufacturer’s instructions. RNA from hippocampal structures was extracted with ReliaPrepTM RNA Miniprep Systems (Promega, Campo Belo, SP, Brazil) according to the manufacturer’s instructions. The RNA samples were quantified in a NanoDrop. The cDNA synthesis was performed with 1 μg of total RNA using the High-Capacity Reverse Transcription Kit with RNase Inhibitor (Thermo Fisher, Somerset, NJ, United States) according to the manufacturer’s instructions in a Master Cycler Gradient thermocycler (Eppendorf, Hamburg, Germany). The RT-qPCR reactions were performed using the Master Mix SYBR Green PCR (Applied Biosystems, Foster City, California, United States) to detect double-stranded DNA synthesis in a QuantStudio™ three Real-Time PCR System (Thermo Fisher Scientific, Somerset, NJ, United States). The reactions were performed in a final volume of 10 μL, using 2 μL of diluted cDNA (1:10) and 300 nM of reverse and forward primers. The comparative cycle threshold (Ct) method (∆∆Ct) was used to calculate the relative gene expression. The β-actin gene (*Actb*) and Gapdh (*Gapdh*) were used as endogenous control. The control group used was the Sham WT. The sequence of primers for amplifying targets used in RT-qPCR was the following: *Ache*, forward: 5′-ACC​TTC​CCT​GGC​TTT​TCC​AC-3′, reverse: 5′-GCA​TCC​AAC​ACT​CCT​GAC​CA-3′; *Iba1*, forward: 5′-GGT​AGA​CAG​TGG​CTT​TCC​CC-3′, reverse: 5′-CTG​TAG​CCC​CTG​AGA​GAG​GT-3′; *Gfap*, forward: 5′-GCC​ACC​AGT​AAC​ATG​CAA​GA-3′, reverse: 5′-GCT​CTA​GGG​ACT​CGT​TCG​TG-3′; *Actb*, forward: 5′-TAT​GCC​AAC​ACA​GTG​CTG​TCT​GG-3′, reverse: 5′-TAC​TCC​TGC​TTG​CTG​ATC​CAC​AT-3′; *Gapdh*, forward: 5′-GGT​CAT​CCC​AGA​GCT​GAA​CG-3′, reverse: 5′-TTG​CTG​TTG​AAG​TCG​CAG​GA-3′.

### 2.6 Western blot analysis

The brain samples (cerebral cortex and hippocampus) were lysed in ice-cold homogenization buffer (50 mM Tris-HCl pH 7.4; 150 mM NaCl; 0.5% Triton X; 1 mM EDTA disodium) supplemented with Protease/Phosphatase inhibitor cocktail (Cell Signaling, Danvers, Massachusetts, United States; #5872S). The lysates were centrifuged at 14,000 × g for 30 min at 4°C. The supernatant was transferred into clean 1.5 ml tubes and stored at −80°C. Protein concentrations were determined by Bio-Rad DC protein assay reagent (Bio-Rad Laboratories), using bovine serum albumin (BSA) as the standard. 30 μg of the total protein was mixed with sample buffer (10 mM Tris-Cl (pH 6.8), 10% glycerol, 1% SDS, 1 mM β-mercaptoethanol and bromophenol blue) and was boiled for 5 min and applied to a 10% SDS-PAGE gel. The proteins separated after migration for 2 h at 120 V were transferred to 0.2 μm nitrocellulose membranes (Bio-Rad Laboratories) at 350 mA for 45 min. Subsequently, the membranes were incubated for 1 h in 5% non-fat milk or 5% BSA in TBS and 0.05% Tween 20 at room temperature. The membranes were incubated overnight at 4°C with primary antibodies diluted in the blocking buffer. Primary antibodies used were anti-GFAP (1:1000) (Cat. No. #3670, Cell Signaling Technology) and anti-β-actin (1:1000) (Cat. No. A1978, Sigma-Aldrich). After overnight incubation, membranes were washed and incubated with HRP-conjugated anti-mouse or anti-rabbit (Thermo Scientific, United States) diluted in TBS-T for 1 h. After another washing step, the membrane was incubated with a peroxidase substrate (SuperSignal West Femto Maximum Sensitivity Substrate) (Thermo Scientific, United States). The bands were visualized by chemiluminescence and quantified by densitometry in ImageJ software.

### 2.7 Acetylcholinesterase activity assay

The AChE assay was performed using brain samples. Cerebral cortex and hippocampus samples were homogenized in a ten-volume solution of 0.1 mM potassium phosphate buffer (pH 7.5). Then, they were centrifuged for 10 min at 1000 × g. The supernatants were used for enzymatic AChE analysis and were determined by a colorimetric method (Ellman et al., 1961). Hydrolysis rates were measured at an acetylthiocholine concentration of 0.8 mM in 300 μL assay solution with 30 mM phosphate buffer (pH 7.5) and 1.0 mM 5,5′-dithiobis-(2-nitrobenzoic acid) (DTNB) (Sigma-Aldrich, St. Louis, MO, United States) at 25°C. Then, 15 μL of cerebral cortex and hippocampus supernatants were added to a reaction mixture and preincubated for 3 min. The formation of the dianion of DTNB by hydrolysis was monitored and measured at 412 nm for 2–3 min intervals of 30 s.

### 2.8 ELISA (enzyme-linked immunosorbent assay)

Brain samples were homogenized in ice-cold phosphate buffer (pH 7.4) (1:4 w/v for cerebral cortex and 1:2 w/v for hippocampus). The homogenate was centrifuged at 800 × g for 5 min at 4°C and the supernatant was used in the analysis to determine the concentration of IL-1β, TNF-α, and IL-10 cytokines, following the manufacturer’s instruction (R&D Systems, Minneapolis, MN, United States). The Bio-Rad (Hercules, CA, United States) protein assay kit was used to determine the protein concentrations.

### 2.9 Data analysis

The results are expressed as mean ± standard error of mean (SEM). Statistical analyses were performed in the object recognition test, molecular and biochemical analyses by one-way analysis of variance (ANOVA), followed by Tukey multiple range tests. The water T-maze test was analyzed by two-way ANOVA followed by Tukey’s post-test. Differences between groups were considered statistically significant when *p* < 0.05.

## 3 Results

### 3.1 P2X7 receptor promotes memory impairment in sepsis-surviving mice

**FIGURE 1:**
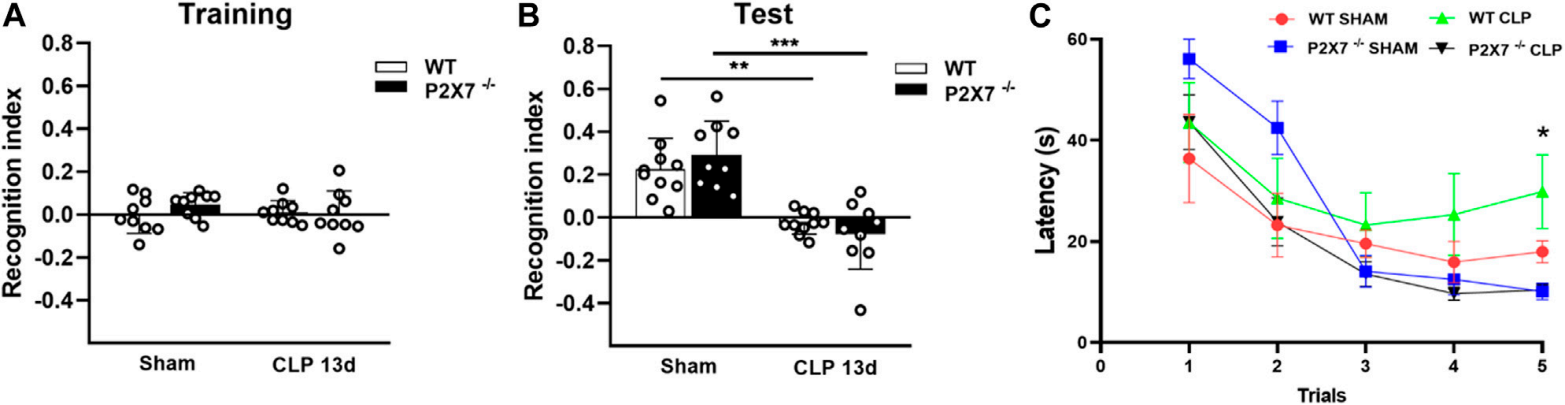
P2X7 receptor contributes to spatial working memory impairment in sepsis-surviving mice 13 days after surgery. Recognition index in the Novel object recognition test (NORT) for CLP (WT and P2X7^−/−^) and sham animals during the training with familiar objects **(A)** and during the test with familiar and novel objects **(B)**. CLP (WT and P2X7^−/−^) and sham mice were subjected to the Water T-maze test (WTMT) **(C)** 13 days post-surgery (* WT CLP vs. P2X7^−/−^ CLP, WT Sham, P2X7^−/−^Sham). Data are expressed as mean ± SEM; One-way ANOVA followed by Tukey’s *post hoc* for recognition index and Two-way ANOVA followed by Tukey’s *post hoc* for WTMT. Asterisks represent statistical differences between groups (*p < 0.05; **p < 0.005; ***p = 0.0001). (*n* = 9–10). The recognition index was calculated by subtracting the time exploring the familiar object from the time exploring the novel object, divided by the total exploration time.

Animals (Sham and CLP-induced mice) were subjected to object recognition and water T-maze tests to evaluate post-sepsis cognitive impairment. We assessed these memory tests to differentiate the brain regions involved in each task. The object recognition test is used to evaluate declarative memory hippocampus-dependent, while the water T-maze test is used to evaluate spatial working memory, which is more pronounced in cortical areas. In the object recognition test, both CLP and Sham animals explored the two familiar objects without significant differences during the training test (*p* > 0.05; Figure 1A). However, a significant decrease in recognition memory index in WT and P2X7^−/−^ septic mice indicated that these animals could not discriminate between novel and familiar objects compared with Sham mice 13 days after CLP surgery. Nevertheless, no significant difference was found between WT and P2X7^−/−^ septic animals (*p* < 0.001; Figure 1B).

When we evaluated the spatial working memory of WT and P2X7^−/−^ mice, we found that post-septic P2X7^−/−^ mice learned the task, but not WT septic mice (*p* < 0.05; Figure 1C). These data suggest that P2X7 receptor is involved in spatial working memory impairment in sepsis-surviving mice, with no impact on recognition memory at the time points evaluated.

As a control, we performed both memory tests in WT and P2X7^−/−^ mice before surgery and found no basal difference between mice strains (Supplementary Figure S1A–D).

### 3.2 P2X7 receptor boosts acetylcholinesterase activity in the brain of sepsis-surviving mice

**FIGURE 2:**
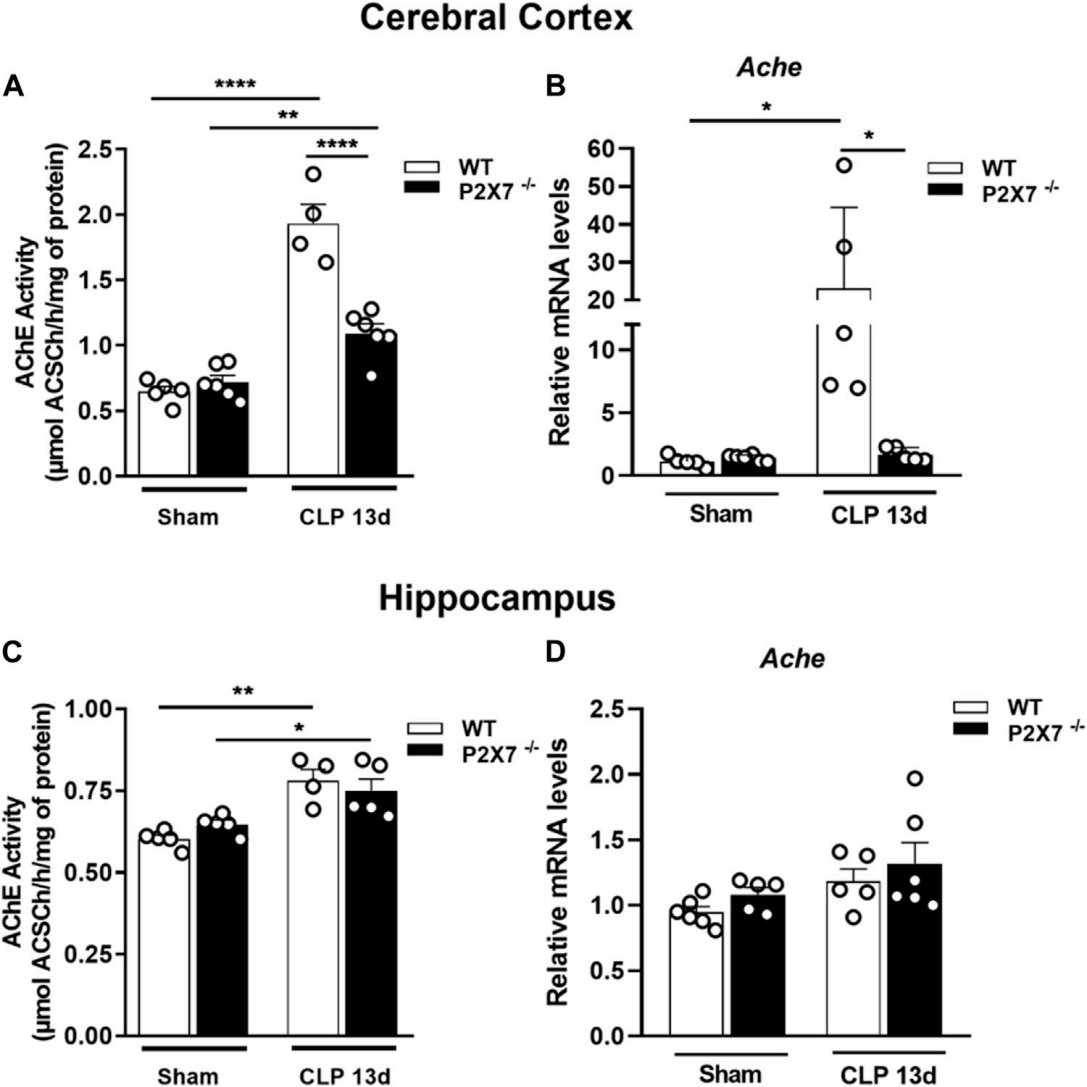
P2X7 receptor functionality increases acetylcholinesterase activity in the cerebral cortex and hippocampus of sepsis-surviving mice. Acetylcholinesterase activity was analyzed by a colorimetric and enzymatic assay and gene expression by RT-qPCR. AChE activity in the cerebral cortex **(A)** and hippocampus **(B)** of CLP and sham mice. Gene expression in the cerebral cortex **(C)** and hippocampus **(D)** Data are expressed as mean ± SEM; One-way ANOVA followed by Tukey’s *post hoc*. Asterisks represent statistical differences between groups (*p < 0.05; **p < 0.001; ****p < 0.0001). Symbols represent individual mice. (*n* = 4–6).

The AChE is responsible for hydrolyzing the neurotransmitter acetylcholine in the synaptic cleft. The increased activity of this enzyme can cause cognitive impairment. We observed an increased AChE activity in the cerebral cortex (*p* < 0.0001 and *p* < 0.05; Figure 2A) and hippocampus (*p* < 0.005 and *p* < 0.05; Figure 2C) in both WT and P2X7^−/−^ post-septic mice. However, P2X7 receptor deletion partially prevented the increase in AChE activity in the cerebral cortex. The AChE activity in this brain structure was significantly lower than in WT mice 13 days after sepsis induction (*p* < 0.0001; Figure 2A).

We detected an increase in AChE mRNA expression in the cerebral cortex of septic mice, while P2X7 receptor deletion prevented this increase (*p* < 0.05; Figure 2B). In the hippocampus, we found no changes in mRNA expression levels (*p* > 0.05; Figure 2D). These results suggest that ATP-P2X7 signaling may contribute to increased AChE activity in sepsis-induced long-term memory.

### 3.3 Genetic blockage of P2X7 receptor prevents long-term neuroinflammation in sepsis-surviving mice

**FIGURE 3:**
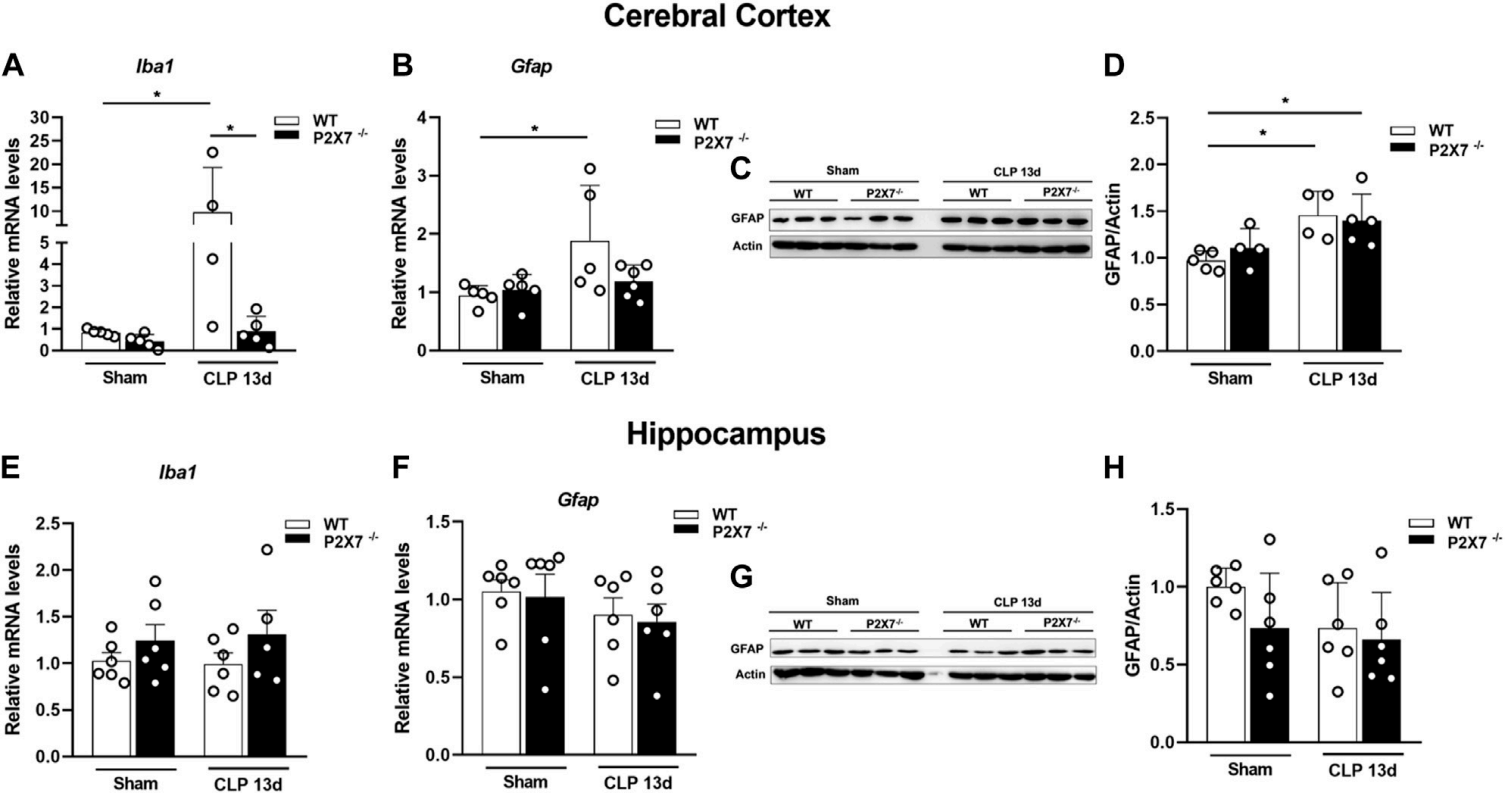
Genetic deletion of P2X7 receptor decreases glial markers Iba-1 and GFAP gene expression in mice cerebral cortex 13 days after surgery. Gene expression Iba-1 and GFAP were analyzed by RT-qPCR and GFAP protein levels were analyzed by Western blotting 13 days after sepsis induction. Iba-1 **(A)** and GFAP **(B)** expression in the cerebral cortex and hippocampus **(E, F)**. Representative Western blot membranes **(C, G)** with densitometric analysis of GFAP **(D, H)**. Data are expressed as mean ± SEM; One-way ANOVA followed by Tukey’s post hoc. Statistical differences between groups are represented by asterisks (*p < 0.05). Symbols represent individual mice. (*n* = 4–6).

The activation of glial cells, such as microglia and astrocytes, characterizes central nervous system (CNS) inflammation. Once activated, these cells can release pro-inflammatory cytokines and reactive oxygen and nitrogen species (ROS and RNS), which promote neuroinflammation, BBB integrity dysfunction, and synaptic loss in brain tissue. Therefore, we evaluated the modulation of glial markers in the brain of sepsis-surviving mice. We found increased mRNA levels of Iba-1 (microglial marker) and GFAP (astrocyte marker) in the cerebral cortex of WT mice but not in the same structure from P2X7^−/−^ septic mice (*p* < 0.05; Figures 3A, B). At the protein level, we detected significantly increased levels of GFAP in the cerebral cortex of both WT and P2X7^−/−^ septic mice at the time point evaluated (*p* < 0.05; Figures 3C, D), indicating the activation of astrocytes. The Iba-1 and GFAP mRNA levels (*p* > 0.05; Figures 3E, F) and GFAP protein expression did not demonstrate differences in the hippocampi of either strain of septic mice at the time point evaluated (*p* > 0.05; Figures 3G, H). These results suggest that the P2X7 receptor might contribute to the long-term expression of glial markers in the cerebral cortex of sepsis-surviving mice.

**FIGURE 4:**
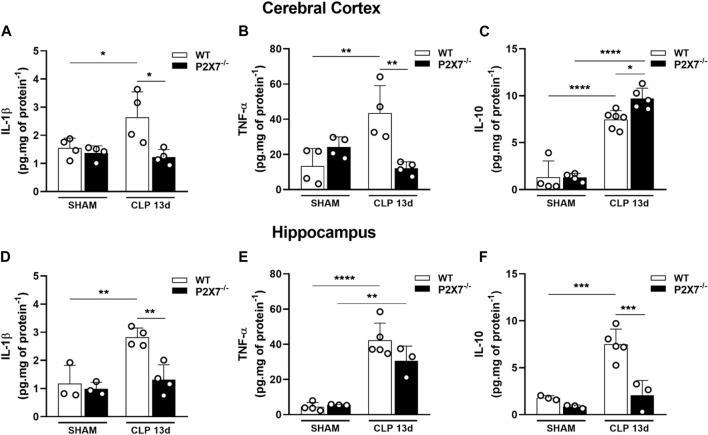
P2X7 receptor genetic deletion reduces cytokine production in the brain of septic-surviving mice. Cytokine production IL-1β, TNF-α, and IL-10 were determined by ELISA in the cerebral cortex **(A–C)**, and hippocampus **(D–F)**. Data are expressed as mean ± SEM; One-way ANOVA followed by Tukey’s *post hoc*. Statistical differences between groups are represented by asterisks (*p < 0.05 **p < 0.005; ***p < 0.0005, ****p < 0.0001). Symbols represent individual mice. (*n* = 3–6).

Although the inflammatory response in sepsis is relatively well understood, the mechanism of cognitive impairment post-sepsis remains unclear. Since septic mice presented cognitive impairment 13 days after sepsis induction and dysregulated cytokine production could be involved in neurological deficits, we evaluated the cytokine production profile in the cerebral cortex and hippocampus of WT and P2X7^−/−^ septic mice. Our results demonstrated an increase in IL-1β and TNF-α in the cerebral cortex of WT but not in P2X7^−/−^ septic mice (*p* < 0.005 and *p* < 0.05; Figures 4A, B). In the hippocampus and cerebral cortex, WT septic mice showed increased IL-1β levels compared to WT sham mice. However, P2X7^−/−^ septic mice did not demonstrate a difference in this parameter compared with P2X7^−/−^ sham. When we compared WT and P2X7^−/−^ septic mice, we observed that the absence of P2X7 receptor prevented this increase. Despite elevated TNF-α production in the hippocampus of WT septic mice, this could not be prevented in P2X7^−/−^septic mice. Increased TNF-α levels were detected in the cerebral cortex of WT septic mice but not in P2X7^−/−^ septic mice. (*p* < 0.05 and *p* < 0.0001; Figures 4D, E).

We investigated the anti-inflammatory cytokine, IL-10, in the cerebral cortex and hippocampus. WT and P2X7^−/−^ septic mice exhibited increased IL-10 levels in the cerebral cortex (*p* < 0.0001; Figure 4C) compared with the sham group. Surprisingly, P2X7^−/−^ septic mice demonstrated that this increase was higher than that in WT septic mice (*p* < 0.05; Figure 4C). WT CLP mice showed an increased production of IL-10 in the hippocampus (*p* < 0.0005; Figure 4F), whereas the absence of the P2X7 receptor in septic mice prevented this increase. These results suggest that sepsis-surviving mice still produce pro-inflammatory cytokines in both brain structures, which could contribute to cognitive impairment. However, P2X7 receptor deletion is important for limiting inflammation in the brains of sepsis-surviving mice.

### 3.4 Pharmacological treatment with a P2X7 receptor inhibitor prevents cognitive impairment and decreased cytokine production in sepsis-surviving mice

**FIGURE 5:**
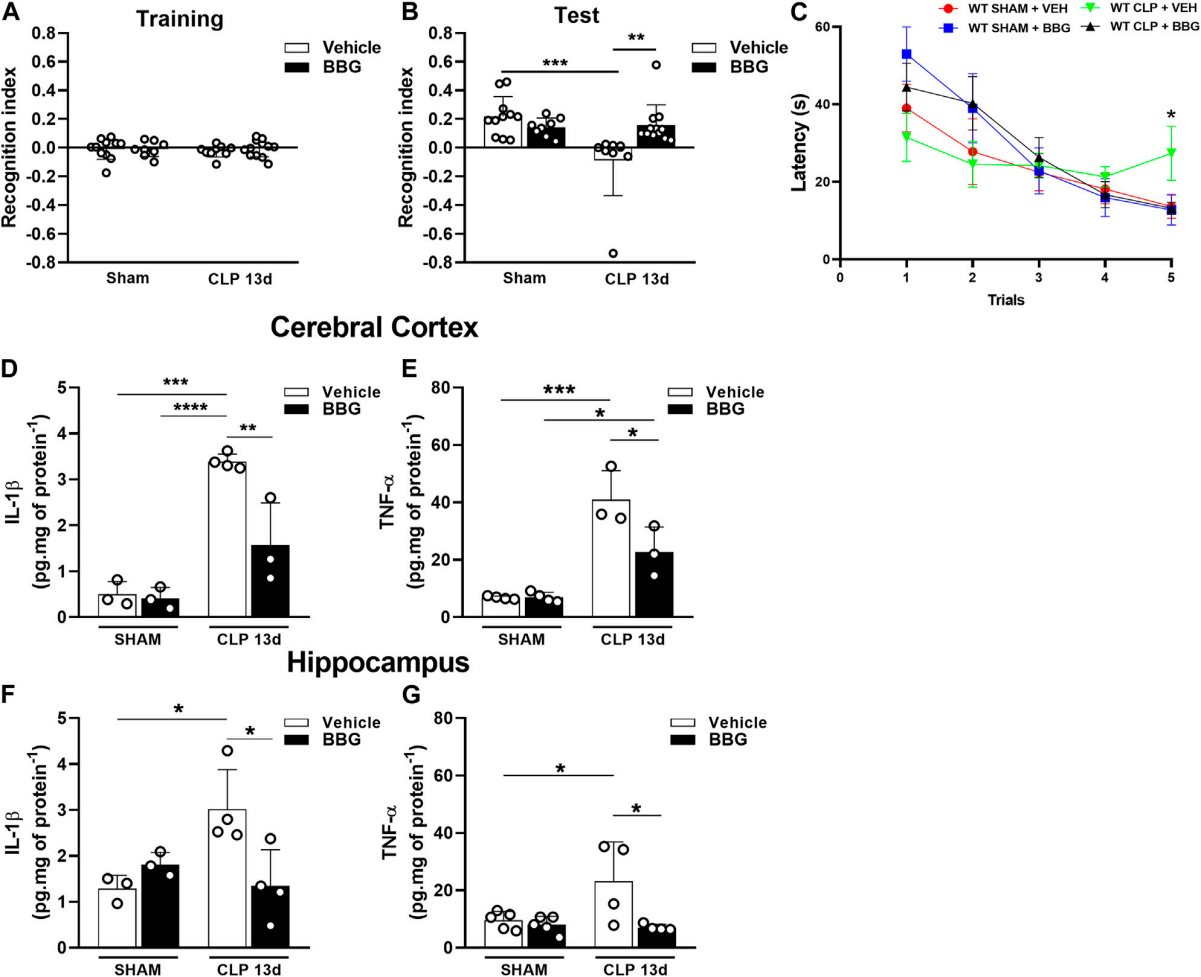
P2X7 receptor blockade with the selective antagonist BBG restored short-term memory impairment and reduced cytokine production in sepsis-surviving mice 13 days after surgery. Recognition index in the Novel object recognition test (NORT) for CLP and sham animals treated with BBG (45 mg/kg) or vehicle (PBS + DMSO 0.2%) 13 days post-surgery during the training with familiar objects **(A)**, and the test with familiar and novel objects **(B)**. CLP and sham animals treated with BBG (45 mg/kg) or vehicle (PBS + DMSO 0.2%) subjected to Water T-maze test (WTMT) **(C)**. Cytokine production IL-1β **(D)** and TNF-α **(E)** in the cerebral cortex, and hippocampus **(F,G)** (*n* = 3–6). Data are expressed as mean ± SEM; One-way ANOVA followed by Tukey’s *post hoc* for recognition index and cytokine production and Two-way ANOVA followed by Tukey’s for WTMT. Statistical differences between groups are represented by asterisks (*p < 0.05; **p < 0.005; ***p < 0.0005). (*n* = 8–12). The recognition index was calculated by subtracting the time exploring the familiar object from the time exploring the novel object, divided by the total exploration time.

We evaluated whether pharmacological blockade of P2X7 receptor with BBG could prevent CLP-induced cognitive impairment in mice 13 days after sepsis. We initially found that BBG or vehicle treatment did not change the animals’ ability to explore familiar objects during training (*p* > 0.05; Figure 5A). Interestingly, BBG treatment prevented cognitive impairment in sepsis-surviving mice compared to that in the vehicle-CLP group when assessed by object recognition test (*p* < 0.0005; Figure 5B). In addition, septic mice treated with BBG learned the task in WTMT compared to vehicle-treated mice, indicating that spatial working memory was preserved with the P2X7 receptor blockade (*p* < 0.05; Figure 5C). Furthermore, P2X7 receptor blockade with BBG decreased IL-1β levels in the cerebral cortex (*p* < 0.0001; Figure 5D) and hippocampus (*p* < 0.05; Figure 5F) in WT septic mice compared to CLP-vehicle mice. BBG treatment decreased TNF-α levels in both cerebral structures compared to vehicle-septic mice (*p* = 0.0001 and *p* < 0.05; Figures 5E, G). These data reinforce the idea that P2X7 blockade may reduce cytokine production and protect against cognitive impairment caused by sepsis.

## 4 Discussion

Sepsis is a life-threatening condition and the leading cause of death in intensive care units (ICU) (Singer et al., 2016; Rudd et al., 2020). Despite the high incidence and mortality worldwide, the number of sepsis survivors over the last few years has increased due to an improvement in supportive care, consisting of antibiotic therapy, hemodynamic management, and supportive care for organ dysfunction during hospitalization (Fleischmann et al., 2016; Prescott and Angus, 2018). After sepsis recovery, patients can develop long-term sequelae, such as immunosuppression, cognitive impairment, fatigue, and depression (Prescott and Angus, 2018). Therefore, a better understanding of these long-term sequelae mechanisms is essential for developing approaches or treatments for long-term cognitive and/or physical disability after hospital discharge (Evans et al., 2021).

Although not well established, the mechanism behind long-term cognitive decline encompasses astrogliosis, microglial activation, compromised BBB integrity, oxidative stress, and changes in brain metabolism (Schwalm et al., 2014; Widmann and Heneka, 2014; Annane and Sharshar, 2015). Considering that under inflammatory conditions, including sepsis, activated and damaged cells release high amounts of ATP, generating a physiologic imbalance that can stimulate the P2 purinergic receptors triggering inflammation (Ledderose et al., 2016), we explored the role of P2X7 receptor on long-term disabilities caused by sepsis in the brain. We found that ATP-P2X7 signaling contributes to neuroinflammation and long-term cognitive dysfunction in sepsis-surviving mice. In addition, antibiotic therapy plus BBG treatment, a P2X7 receptor blocker, prevented these deleterious effects and improved sepsis outcomes.

Behavioral impairment has been established as a consequence of sepsis. Previous reports demonstrated that worsening learning and memory do not follow a specific time course and might occur from 24 h up to full recovery 60 days post-sepsis, depending on the sepsis model [e.g. CLP; lipopolysaccharide (LPS)], animal type, and severity (Shimizu et al., 1999; Barichello et al., 2005, 2007; Tuon et al., 2008; Belikoff et al., 2011; Anderson et al., 2015; Neves et al., 2018; De Sousa et al., 2021). The object recognition test is an efficient way to identify memory changes in mice, as object preference and distinction are used as indicators of changed behavior (Akkerman et al., 2012; Leger et al., 2013). Recognition memory is hippocampus-dependent (Barker and Warburton, 2011; Stanley et al., 2012), and lesions in this region impair recognition memory (Clark et al., 2000). Loss of short-term memory and spatial recognition indicate that sepsis compromises the brain (Ebersoldt et al., 2007). Besides, the hippocampus is vulnerable and susceptible to inflammation due to sepsis (Lynch et al., 2004; Annane, 2009). The water T-maze is a method used to assess spatial working memory, and its main advantage is that it avoids the negative reinforcement present in the T-maze or Morris water tasks (Locchi et al., 2007). Cortical regions of the brain are implicated in spatial working memory, mainly the prefrontal and perirhinal cortices (Romanides et al., 1999; Maioli et al., 2012). It has been reported that sepsis compromises spatial learning and working memory (Yu et al., 2014; Li et al., 2017; Wang et al., 2022).

We observed that 13 days after surgery, septic-surviving WT mice presented impaired declarative memory, which is in accordance with several studies that demonstrated this impairment at 7, 9, and 10 days after CLP (Barichello et al., 2007; Cassol-Jr et al., 2011; Petronilho et al., 2012; Huang et al., 2014; Calsavara et al., 2015; Moraes et al., 2015). In addition, P2X7^−/−^ septic mice exhibited recognition memory impairment, as demonstrated by a decreased recognition index (NORT). In contrast, BBG treatment prevented this impairment, indicating that pharmacological treatment may more effectively prevent sepsis-induced cognitive dysfunctions in the evaluated time. Differently, the P2X7 receptor genetic ablation and blockade preserved spatial working memory (MWMT) after sepsis, corroborating the findings of Wang et al. (2022) who demonstrated that P2X7 blockade improved spatial memory in sepsis-surviving mice 7 days after sepsis induction. P2X7 receptor deletion or inhibition improves behavioral changes in certain neurological diseases, such as Alzheimer’s disease, Huntington’s disease, and stress conditions (Díaz-Hernández et al., 2009; Iwata et al., 2016; Farooq et al., 2018; Martin et al., 2019). Furthermore, the administration of BBG reversed cognitive deficits induced by Aβ (amyloid protein) in an Alzheimer’s model (Chen et al., 2014). Interestingly, there are conflicting data about the P2X7 receptor in behavioral tests, which can be related to the P2X7 gene proximity to the locus for affective disorders (Lucae et al., 2006). Labrousse et al. (2009) demonstrated impaired spatial memory in the Y-Maze test but not in recognition memory performance in P2X7 knockout mice. We did not detect any changes in behavioral tests of P2X7 receptor-knockout mice under basal conditions.

Cholinergic pathway hypofunction is related to behavioral abnormalities (Ricceri et al., 2004). It has been proposed that sepsis might alter cholinergic signaling, and once after sepsis recovery, animals present long-term cognitive impairment, which is attributed to reduced cholinergic innervation (Semmler et al., 2007; van Gool et al., 2010). In addition, this pathway may exert an anti-inflammatory effect by suppressing the release of pro-inflammatory cytokines during endotoxemia (Borovikova et al., 2000). Overexpression of human AChE in transgenic mice leads to cognitive impairment (Beeri et al., 1995). Considering the relation between sepsis and the cholinergic pathway, we evaluated AChE activity in the brain of sepsis-surviving mice. We observed a significant increase in AChE activity in the hippocampus and cerebral cortex of both septic-surviving mice (WT and P2X7^−/−^). Corroborating with these findings, Zaghloul and others demonstrated that septic-surviving mice presented increased AChE activity in the hippocampus and cerebral cortex (Zaghloul et al., 2017). In addition, IL-1β induced AChE mRNA expression *in vivo* and activity *in vitro* (Li et al., 2017), reflecting the pro-inflammatory scenario caused by sepsis. However, P2X7^−/−^ septic mice demonstrated decreased AChE activity and expression in the cerebral cortex compared to WT septic mice. It has been reported that septic-surviving mice treated with cholinesterase inhibitors presented lower inflammation (Jeremias et al., 2016). Therefore, our results could partly explain that the memory impairment detected in NORT depends on increased AChE activity in the hippocampus, while the preserved spatial working memory in the WTMT in P2X7^−/−^ septic mice might be due to a reduced AChE activity in cerebral cortex compared to WT septic mice. Indeed, low levels of acetylcholine may negatively modulate memory, which correlates with high levels of AChE (Soreq, 2001), and microglial activation has been suggested to dysregulate cholinergic components (Zaghloul et al., 2017). Furthermore, AChE can activate glial cells promoting an inflammatory response in AD models (Von Bernhardi et al., 2003).

The blood-brain barrier is disrupted by systemic inflammation, allowing inflammatory mediators to enter the brain and activate microglial cells (Sankowski et al., 2015). Glial activation seems to be chronic in sepsis, since 48 h and 10 days later, it continues to occur in the hippocampus and prefrontal cortex (Catalão et al., 2017, 2020). Accordingly, we found that positive modulation of glial markers continued to occur in the cerebral cortex 13 days after sepsis induction. We found that Iba1 and GFAP were upregulated in WT septic mice, whereas P2X7^−/−^ septic mice prevented this event in the cerebral cortex, indicating that the absence of the P2X7 receptor may be involved in the reversal GFAP and Iba-1 upregulation during sepsis. In addition, GFAP expression increased at the protein level in both CLP groups indicating astrocytic activation. However, in P2X7^−/−^ septic mice, GFAP gene expression cannot be correlated with increased protein levels because of the several mechanisms of gene expression regulation and translation or post-translational events that might occur during distinct stimuli, such as injury and cellular stress, among others (Escartin and Galea, 2021). Indeed, even without disease, the GFAP levels are fluid and may vary from cell to cell. Therefore, future studies investigating inflammatory signals that promote astrocyte polarization and phenotypic changes are also required (Liddelow and Barres, 2017; Lawrence et al., 2023). Surprisingly, we did not detect an increase in the expression of these glial markers in the hippocampus during this period. Some studies have pointed out that astrocytic activation can persist for up to 15 days after sepsis onset, depending on the sepsis model (Danielski et al., 2022). Furthermore, Hernandes and others demonstrated that microglia and astrocytes were activated 5 days after the onset of sepsis in the hippocampus (Hernandes et al., 2014), reinforcing the complexity of sepsis and the experimental model used. Long-term cognitive dysfunction in this context also depends on microglial reactivity and inflammatory processes due to sepsis (Michels et al., 2015).

Under systemic inflammation, eATP is increased and reaches the brain being able to activate the P2X7 receptor (Virgilio et al., 2023). In addition, the activated glial cell can secrete ATP in brain tissue. Once activated, the P2X7 receptor acts as a second signal for the inflammasome assembly, caspase-1 activation, and IL-1β cleavage (Piccini et al., 2008). A previous study by our group showed that IL-1β levels were increased in the hippocampus and cerebral cortex of WT septic mice 24 h after sepsis (Savio et al., 2017). Cultured mouse primary microglia have been described as the cells responsible for IL-1β release, and this mechanism is dependent on P2X7 receptor activation, in contrast to TNF-α (Mingam et al., 2008; Shieh et al., 2014). Moraes and others verified microglial and astrocytic activation in the brain of mice 24 h after sepsis induction, attributing the main source of cognitive deficit to IL-1β released from microglia (Moraes et al., 2015). We observed a sustained neuroinflammatory process in these surviving animals, and 13 days after sepsis induction, we detected increased IL-1β and TNF-α levels in both structures (hippocampus and cerebral cortex) of septic-surviving WT mice. We also found that genetic deletion or pharmacological blockade (BBG) of the P2X7 receptor in septic-surviving mice decreased the level of these cytokines in both structures 13 days after sepsis, demonstrating that this receptor can contribute to the neuroinflammatory process in sepsis survivors. In the same line of evidence, it was demonstrated that P2X7 receptor inhibition limited the inflammatory response induced by LPS in microglial cells (Choi et al., 2007). In addition, P2X7 blockade with BBG decreases TNF-α levels after LPS injection (Ma et al., 2014), and IL-1β in the cerebral cortex and hippocampus (Savio et al., 2017). The ability of Brilliant Blue G to penetrate the blood-brain barrier exerts a protective effect in certain neuropathologies, such as spinal cord injury and Alzheimer’s (Peng et al., 2009; Diaz-Hernandez et al., 2012), possibly explaining our results of the decreased cytokine levels and behavioral improvement. Despite the selectivity for the P2X7 receptor, BBG can inhibit, to a lesser extent, the P2X4 receptor (Jiang et al., 2000); therefore, we cannot rule out additional effects in cognitive improvement in long-term neurological alterations after sepsis.

Here, we found increased levels of IL-10 in the cerebral cortex of both CLP mice (WT and P2X7^−/−^). Despite increased IL-10 levels in the hippocampus of WT septic mice, genetic deletion of P2X7 decreased this cytokine. This result partially corroborates our previous work, which demonstrated that P2X7 inhibition decreased IL-10 levels in the hippocampus 24 h after sepsis induction (Savio et al., 2017). Furthermore, it has been demonstrated that septic mice survivors present high levels of IL-10 in the serum, which correlates with the coexistence of the compensatory state in sepsis survivors (Zaghloul et al., 2017). In fact, reports demonstrate that IL-10 prevents neurodegeneration in the cerebral cortex of LPS-induced rats and can limit the neuroinflammation caused by CNS pathogens (Rasley et al., 2006; Keun et al., 2007).

Taken together, our data suggest that ATP-P2X7R axis contributed to exacerbating the harmful effects caused by sepsis in the brain since the absence or blockade of P2X7 receptor improved cognitive impairment and modulated sepsis-associated neuroinflammation. Thus, these results suggest using the P2X7 receptor as a pharmacological target in adjuvant therapies to modulate sepsis-associated brain dysfunction.

## Data Availability

The raw data supporting the conclusion of this article will be made available by the authors, without undue reservation.

## References

[B1] Akkerman S. Blokland A. Reneerkens O. van Goethem N. P. Bollen E. Gijselaers H. J. M. (2012). Object recognition testing: Methodological considerations on exploration and discrimination measures. Behav. Brain Res. 232, 335–347. 10.1016/j.bbr.2012.03.022 22490364

[B2] Anderson S. T. Commins S. Moynagh P. N. Coogan A. N. (2015). Lipopolysaccharide-induced sepsis induces long-lasting affective changes in the mouse. Brain. Behav. Immun. 43, 98–109. 10.1016/j.bbi.2014.07.007 25063709

[B3] Annane D. (2009). Hippocampus: A future target for sepsis treatment. Intensive Care Med. 35, 585–586. 10.1007/s00134-009-1395-6 19156398

[B4] Annane D. Sharshar T. (2015). Cognitive decline after sepsis. Lancet Respir. Med. 3, 61–69. 10.1016/S2213-2600(14)70246-2 25434614

[B5] Baker C. C. Chaudry I. H. Gaines H. O. Baue A. E. (1983). Evaluation of factors affecting mortality rate after sepsis in a murine cecal ligation and puncture model. Surgery 94, 331–335. 6879447

[B6] Barichello T. Martins M. R. Reinke A. Constantino L. S. Machado R. A. Valvassori S. S. (2007). Behavioral deficits in sepsis-surviving rats induced by cecal ligation and perforation. Braz. J. Med. Biol. Res. 40, 831–837. 10.1590/S0100-879X2007000600013 17581683

[B7] Barichello T. Martins M. R. Reinke A. Feier G. Ritter C. Quevedo J. (2005). Cognitive impairment in sepsis survivors from cecal ligation and perforation. Crit. Care Med. 33, 221–223. 10.1097/01.CCM.0000150741.12906 15644673

[B8] Barker G. R. I. Warburton E. C. (2011). When is the hippocampus involved in recognition memory? J. Neurosci. 31, 10721–10731. 10.1523/JNEUROSCI.6413-10.2011 21775615 PMC6622630

[B9] Beeri R. Andres C. Lev-Lehman E. Timberg R. Huberman T. Shani M. (1995). Transgenic expression of human acetylcholinesterase induces progressive cognitive deterioration in mice. Curr. Biol. 5, 1063–1071. 10.1016/S0960-9822(95)00211-9 8542283

[B10] Belikoff B. G. Hatfield S. Georgiev P. Ohta A. Lukashev D. Buras J. A. (2011). A2B adenosine receptor blockade enhances macrophage-mediated bacterial phagocytosis and improves polymicrobial sepsis survival in mice. J. Immunol. 186, 2444–2453. 10.4049/jimmunol.1001567 21242513 PMC3708265

[B11] Borovikova L. V. Ivanova S. Zhang M. Yang H. Botchkina G. I. Watkins L. R. (2000). Vagus nerve stimulation attenuates the systemic inflammatory response to endotoxin. Nature 405, 458–462. 10.1038/35013070 10839541

[B12] Calsavara A. C. Soriani F. M. Vieira L. Q. Costa P. A. Rachid M. A. Teixeira A. L. (2015). TNFR1 absence protects against memory deficit induced by sepsis possibly through over-expression of hippocampal BDNF. Metab. Brain Dis. 30, 669–678. 10.1007/s11011-014-9610-8 25148914

[B13] Cassol-O. J. Jr Comim C. M. Constantino L. S. Rosa D. V. F. Mango L. A. V. Stertz L. (2011). Acute low dose of MK-801 prevents memory deficits without altering hippocampal DARPP-32 expression and BDNF levels in sepsis survivor rats. J. Neuroimmunol. 230, 48–51. 10.1016/j.jneuroim.2010.08.026 20864183

[B14] Catalão C. H. R. Santos-Júnior N. N. da Costa L. H. A. Souza A. O. Alberici L. C. Rocha M. J. A. (2017). Brain oxidative stress during experimental sepsis is attenuated by simvastatin administration. Mol. Neurobiol. 54, 7008–7018. 10.1007/s12035-016-0218-3 27796742

[B15] Catalão C. H. R. Santos-Junior N. N. da Costa L. H. A. Souza A. O. Cárnio E. C. Sebollela A. (2020). Simvastatin prevents long-term cognitive deficits in sepsis survivor rats by reducing neuroinflammation and neurodegeneration. Neurotox. Res. 38, 871–886. 10.1007/s12640-020-00222-z 32524380

[B16] Cauwels A. Rogge E. Vandendriessche B. Shiva S. Brouckaert P. (2014). Extracellular ATP drives systemic inflammation, tissue damage and mortality. Cell Death Dis. 5, e1102. 10.1038/cddis.2014.70 24603330 PMC3973196

[B17] Cavaillon J. Singer M. Skirecki T. (2020). Sepsis therapies: Learning from 30 years of failure of translational research to propose new leads. EMBO Mol. Med. 12, 101288–e10224. 10.15252/emmm.201810128 PMC7136965 32176432

[B18] Chen X. Hu J. Jiang L. Xu S. Zheng B. Wang C. (2014). Brilliant Blue G improves cognition in an animal model of Alzheimer’s disease and inhibits amyloid-β-induced loss of filopodia and dendrite spines in hippocampal neurons. Neuroscience 279, 94–101. 10.1016/j.neuroscience.2014.08.036 25193238

[B19] Choi H. B. Ryu J. K. Kim S. U. McLarnon J. G. (2007). Modulation of the purinergic P2X7 receptor attenuates lipopolysaccharide-mediated microglial activation and neuronal damage in inflamed brain. J. Neurosci. 27, 4957–4968. 10.1523/JNEUROSCI.5417-06.2007 17475804 PMC6672082

[B20] Chung H.-Y. Wickel J. Brunkhorst F. M. Geis C. (2020). Sepsis-associated encephalopathy: From delirium to dementia? J. Clin. Med. 9, 703. 10.3390/jcm9030703 32150970 PMC7141293

[B21] Clark R. E. Zola S. M. Squire L. R. (2000). Impaired recognition memory in rats after damage to the hippocampus. J. Neurosci. 20, 8853–8860. 10.1523/jneurosci.20-23-08853.2000 11102494 PMC6773055

[B22] Csóka B. Németh Z. H. Szabó I. Davies D. L. Varga Z. V. Pálóczi J. (2018). Macrophage P2X4 receptors augment bacterial killing and protect against sepsis. JCI insight 3, 994311–e99518. 10.1172/jci.insight.99431 PMC5997389 29875325

[B23] Danielski L. G. Giustina A. Della Gava F. F. Barichello T. Petronilho F. (2022). The many faces of astrocytes in the septic brain. Mol. Neurobiol. 59, 7229–7235. 10.1007/s12035-022-03027-7 36136265

[B24] De Sousa V. L. Araújo S. B. Antonio L. M. Silva-Queiroz M. Colodeti L. C. Soares C. (2021). Innate immune memory mediates increased susceptibility to Alzheimer’s disease-like pathology in sepsis surviving mice. Brain. Behav. Immun. 95, 287–298. 10.1016/j.bbi.2021.04.001 33838250

[B25] Di Virgilio F. Dal Ben D. Sarti A. C. Giuliani A. L. Falzoni S. (2017). The P2X7 receptor in infection and inflammation. Immunity 47, 15–31. 10.1016/j.immuni.2017.06.020 28723547

[B26] Di Virgilio F. Sarti A. C. Coutinho-Silva R. (2020). Purinergic signaling, DAMPs, and inflammation. Am. J. Physiol. - Cell Physiol. 318, C832–C835. 10.1152/ajpcell.00053.2020 32159362

[B27] Diaz-Hernandez J. I. Gomez-Villafuertes R. León-Otegui M. Hontecillas-Prieto L. del Puerto A. Trejo J. L. (2012). *In vivo* P2X7 inhibition reduces amyloid plaques in Alzheimer’s disease through GSK3β and secretases. Neurobiol. Aging 33, 1816–1828. 10.1016/j.neurobiolaging.2011.09.040 22048123

[B28] Díaz-Hernández M. Díez-Zaera M. Sánchez-Nogueiro J. Gómez-Villafuertes R. Canals J. M. Alberch J. (2009). Altered P2X7-receptor level and function in mouse models of Huntington’s disease and therapeutic efficacy of antagonist administration. FASEB J. 23, 1893–1906. 10.1096/fj.08-122275 19171786

[B29] Ebersoldt M. Sharshar T. Annane D. (2007). Sepsis-associated delirium. Intensive Care Med. 33, 941–950. 10.1007/s00134-007-0622-2 17410344

[B30] Ellman G. L. Courtney K. D. Andres V. Featherstone R. M. (1961). A new and rapid colorimetric determination of acetylcholinesterase activity. Biochem. Pharmacol. 7, 88–95. 10.1016/0006-2952(61)90145-9 13726518

[B31] Escartin C. Galea E. Lakatos A. O'Callaghan J. P. Petzold G. C. Serrano-Pozo A. (2021). Reactive astrocyte nomenclature, definitions, and future directions. Nat. Neurosci. 24, 312–325. 10.1038/s41593-020-00783-4 33589835 PMC8007081

[B32] Evans L. Rhodes A. Alhazzani W. Antonelli M. Coopersmith C. M. French C. (2021). Surviving sepsis campaign: International guidelines for management of sepsis and septic shock 2021. Intensive Care Med. 47, 1181–1247. 10.1007/s00134-021-06506-y 34599691 PMC8486643

[B33] Farooq R. K. Tanti A. Ainouche S. Roger S. Belzung C. Camus V. (2018). A P2X7 receptor antagonist reverses behavioural alterations, microglial activation and neuroendocrine dysregulation in an unpredictable chronic mild stress (UCMS) model of depression in mice. Psychoneuroendocrinology 97, 120–130. 10.1016/j.psyneuen.2018.07.016 30015007

[B34] Fleischmann C. Scherag A. Adhikari N. K. J. Hartog C. S. Tsaganos T. Schlattmann P. (2016). Assessment of global incidence and mortality of hospital-treated sepsis current estimates and limitations. Am. J. Respir. Crit. Care Med. 193, 259–272. 10.1164/rccm.201504-0781OC 26414292

[B35] Fredholm B. B. Ijzerman A. P. Jacobson K. A. Linden J. Müller C. E. (2011). International union of basic and clinical pharmacology. LI. Nomenclature and classification of adenosine receptors - an update. Pharmacol. Rev. 63, 1–34. 10.1124/pr.110.003285 21303899 PMC3061413

[B36] Geistlinger J. Du W. Groll J. Liu F. Hoegel J. Foehr K. J. (2012). P2RX7 genotype association in severe sepsis identified by a novel Multi-Individual Array for rapid screening and replication of risk SNPs. Clin. Chim. Acta 413, 39–47. 10.1016/j.cca.2011.05.023 21640086

[B94] Hernandes M. S. D'Avila J. C. Trevelin S. C. Reis P. A. Kinjo E. R. Lopes L. R. (2014). The role of Nox2-derived ROS in the development of cognitive impairment after sepsis. J Neuroinflammation 11, 36. 10.1186/1742-2094-11-36 24571599 PMC3974031

[B37] Huang M. Liu C. H. Hu Y. Y. Wang P. F. Ding M. P. (2014). γ-secretase inhibitor DAPT prevents neuronal death and memory impairment in sepsis associated encephalopathy in septic rats. Chin. Med. J. Engl. 127, 924–928. 10.3760/cma.j.issn.0366-6999.20132366 24571889

[B38] Idzko M. Ferrari D. Eltzschig H. K. (2014). Nucleotide signalling during inflammation. Nature 509, 310–317. 10.1038/nature13085 24828189 PMC4222675

[B39] Illes P. Müller C. E. Jacobson K. A. Grutter T. Nicke A. Fountain S. J. (2020). Update of P2X receptor properties and their pharmacology: IUPHAR review 30. Br. J. Pharmacol. 178, 489–514. 10.1111/bph.15299 33125712 PMC8199792

[B40] Iwashyna T. J. Ely E. W. Smith D. M. Langa K. M. (2010). Long-term cognitive impairment and functional disability among survivors of severe sepsis. JAMA 304, 1787–1794. 10.1001/jama.2010.1553 20978258 PMC3345288

[B41] Iwata M. Ota K. T. Li X. Y. Sakaue F. Li N. Dutheil S. (2016). Psychological stress activates the inflammasome via release of adenosine triphosphate and stimulation of the purinergic type 2X7 receptor. Biol. Psychiatry 80, 12–22. 10.1016/j.biopsych.2015.11.026 26831917

[B42] Jeremias I. C. Victorino V. J. Barbeiro H. V. Kubo S. A. Prado C. M. Lima T. M. (2016). The role of acetylcholine in the inflammatory response in animals surviving sepsis induced by cecal ligation and puncture. Mol. Neurobiol. 53, 6635–6643. 10.1007/s12035-015-9538-y 26637327

[B43] Jiang L.-H. Mackenzie A. B. North R. A. Surprenant A. (2000). Brilliant blue G selectively blocks ATP-gated rat P2X 7 receptors. Mol. Pharmacol. 58, 82–88. 10.1124/mol.58.1.82 10860929

[B44] Keun W. P. Hwan G. L. Byung K. J. Yong B. L. (2007). Interleukin-10 endogenously expressed in microglia prevents lipopolysaccharide-induced neurodegeneration in the rat cerebral cortex *in vivo* . Exp. Mol. Med. 39, 812–819. 10.1038/emm.2007.88 18160852

[B45] Labrousse V. F. Costes L. Aubert A. Darnaudéry M. Ferreira G. Amédée T. (2009). Impaired interleukin-1beta and c-Fos expression in the hippocampus is associated with a spatial memory deficit in P2X(7) receptor-deficient mice. PLoS One 4, e6006. 10.1371/journal.pone.0006006 19547756 PMC2695542

[B46] Lawrence J. M. Schardien K. Wigdahl B. Nonnemacher M. R. (2023). Roles of neuropathology-associated reactive astrocytes: A systematic review. Acta Neuropathol. Commun. 111 11, 42–28. 10.1186/s40478-023-01526-9 PMC10009953 36915214

[B47] Ledderose C. Bao Y. Kondo Y. Fakhari M. Slubowski C. Zhang J. (2016). Purinergic signaling and the immune response in sepsis: A review. Clin. Ther. 38, 1054–1065. 10.1016/j.clinthera.2016.04.002 27156007 PMC4875817

[B48] Leger M. Quiedeville A. Bouet V. Haelewyn B. Boulouard M. Schumann-Bard P. (2013). Object recognition test in mice. Nat. Protoc. 8, 2531–2537. 10.1038/nprot.2013.155 24263092

[B49] Leite-Aguiar R. Alves V. S. Savio L. E. B. Coutinho-Silva R. (2021). Targeting purinergic signaling in the dynamics of disease progression in sepsis. Front. Pharmacol. 11, 626484–626486. 10.3389/fphar.2020.626484 33519492 PMC7840482

[B50] Li Y. Wang F. Luo Y. (2017). Ginsenoside Rg1 protects against sepsis-associated encephalopathy through beclin 1–independent autophagy in mice. J. Surg. Res. 207, 181–189. 10.1016/j.jss.2016.08.080 27979475

[B51] Liddelow S. A. Barres B. A. (2017). Reactive astrocytes: Production, function, and therapeutic potential. Immunity 46, 957–967. 10.1016/j.immuni.2017.06.006 28636962

[B52] Locchi F. Dall’Olio R. Gandolfi O. Rimondini R. (2007). Water T-maze, an improved method to assess spatial working memory in rats: Pharmacological validation. Neurosci. Lett. 422, 213–216. 10.1016/j.neulet.2007.06.023 17629404

[B53] Lucae S. Salyakina D. Barden N. Harvey M. Gagné B. Labbé M. (2006). P2RX7, a gene coding for a purinergic ligand-gated ion channel, is associated with major depressive disorder. Hum. Mol. Genet. 15, 2438–2445. 10.1093/hmg/ddl166 16822851

[B54] Lynch A. M. Walsh C. Delaney A. Nolan Y. Campbell V. A. Lynch M. A. (2004). Lipopolysaccharide-induced increase in signalling in hippocampus is abrogated by IL-10 - a role for IL-1β? J. Neurochem. 88, 635–646. 10.1046/j.1471-4159.2003.02157.x 14720213

[B55] Ma M. Ren Q. Zhang J. C. Hashimoto K. (2014). Effects of brilliant blue G on serum tumor necrosis factor-α levels and depression-like behavior in mice after lipopolysaccharide administration. Clin. Psychopharmacol. Neurosci. 12, 31–36. 10.9758/cpn.2014.12.1.31 24851118 PMC4022763

[B56] Maioli S. Gangarossa G. Locchi F. Andrioli A. Bertini G. Rimondini R. (2012). Excitotoxic lesion of the perirhinal cortex impairs spatial working memory in a delayed-alternation task. Behav. Brain Res. 230, 349–354. 10.1016/j.bbr.2012.02.030 22391121

[B57] Martin E. Amar M. Dalle C. Youssef I. Boucher C. Le Duigou C. (2019). New role of P2X7 receptor in an Alzheimer’s disease mouse model. Mol. Psychiatry 24, 108–125. 10.1038/s41380-018-0108-3 29934546 PMC6756107

[B58] Martínez-García J. J. Martínez-Banaclocha H. Angosto-Bazarra D. de Torre-Minguela C. Baroja-Mazo A. Alarcón-Vila C. (2019). P2X7 receptor induces mitochondrial failure in monocytes and compromises NLRP3 inflammasome activation during sepsis. Nat. Commun. 10, 2711. 10.1038/s41467-019-10626-x 31221993 PMC6586640

[B59] Michels M. Vieira A. S. Vuolo F. Zapelini H. G. Mendonça B. Mina F. (2015). The role of microglia activation in the development of sepsis-induced long-term cognitive impairment. Brain. Behav. Immun. 43, 54–59. 10.1016/j.bbi.2014.07.002 25019583

[B60] Mingam R. Smedt V. D. Amédée T. Bluthé R. M. Kelley K. W. Dantzer R. (2008). *In vitro* and *in vivo* evidence for a role of the P2X7 receptor in the release of IL-1β in the murine brain. Brain. Behav. Immun. 22, 234–244. 10.1016/j.bbi.2007.08.007 17905568 PMC2908086

[B61] Moraes C. A. Santos G. Spohr T. C. L. S. D’Avila J. C. Lima F. R. S. Benjamim C. F. (2015). Activated microglia-induced deficits in excitatory synapses through IL-1β: Implications for cognitive impairment in sepsis. Mol. Neurobiol. 52, 653–663. 10.1007/s12035-014-8868-5 25257696

[B62] Neves F. S. Marques P. T. Barros-Aragão F. Nunes N. B. Venancio A. M. Cozachenco D. (2018). Brain-defective insulin signaling is associated to late cognitive impairment in post-septic mice. Mol. Neurobiol. 55, 435–444. 10.1007/s12035-016-0307-3 27966074

[B63] Nikayin S. Rabiee A. Hashem M. D. Huang M. Bienvenu O. J. Turnbull A. E. (2016). Anxiety symptoms in survivors of critical illness: A systematic review and meta-analysis. Gen. Hosp. Psychiatry 43, 23–29. 10.1016/j.genhosppsych.2016.08.005 27796253 PMC5289740

[B64] Peng W. Cotrina M. L. Han X. Yu H. Bekar L. Blum L. (2009). Systemic administration of an antagonist of the ATP-sensitive receptor P2X7 improves recovery after spinal cord injury. Proc. Natl. Acad. Sci. U. S. A. 106, 12489–12493. 10.1073/pnas.0902531106 19666625 PMC2718350

[B65] Petronilho F. Périco S. R. Vuolo F. Mina F. Constantino L. Comim C. M. (2012). Protective effects of guanosine against sepsis-induced damage in rat brain and cognitive impairment. Brain. Behav. Immun. 26, 904–910. 10.1016/j.bbi.2012.03.007 22497789

[B66] Piccini A. Carta S. Tassi S. Lasiglié D. Fossati G. Rubartelli A. (2008). ATP is released by monocytes stimulated with pathogen-sensing receptor ligands and induces IL-1beta and IL-18 secretion in an autocrine way. Proc. Natl. Acad. Sci. U. S. A. 105, 8067–8072. 10.1073/pnas.0709684105 18523012 PMC2430360

[B67] Prescott H. C. Angus D. C. (2018). Enhancing recovery from sepsis: A review. JAMA - J. Am. Med. Assoc. 319, 62–75. 10.1001/jama.2017.17687 PMC5839473 29297082

[B68] Rabiee A. Nikayin S. Hashem M. D. Huang M. Dinglas V. D. Bienvenu O. J. (2016). Depressive symptoms after critical illness: A systematic review and meta-analysis. Crit. Care Med. 44, 1744–1753. 10.1097/CCM.0000000000001811 27153046 PMC7418220

[B69] Rasley A. Tranguch S. L. Rati D. M. Marriott I. (2006). Murine glia express the immunosuppressive cytokine, interleukin-10, following exposure toBorrelia burgdorferi orNeisseria meningitidis. Glia 53, 583–592. 10.1002/glia.20314 16419089

[B70] Reinhart K. Daniels R. Kissoon N. Machado F. R. Schachter R. D. Finfer S. (2017). Recognizing sepsis as a global health priority — a WHO resolution. N. Engl. J. Med. 377, 414–417. 10.1056/NEJMp1707170 28658587

[B71] Ricceri L. Minghetti L. Moles A. Popoli P. Confaloni A. De Simone R. (2004). Cognitive and neurological deficits induced by early and prolonged basal forebrain cholinergic hypofunction in rats. Exp. Neurol. 189, 162–172. 10.1016/j.expneurol.2004.05.025 15296846

[B72] Rittirsch D. Huber-Lang M. S. Flierl M. A. Ward P. A. (2009). Immunodesign of experimental sepsis by cecal ligation and puncture. Nat. Protoc. 4, 31–36. 10.1038/nprot.2008.214 19131954 PMC2754226

[B73] Romanides A. J. Duffy P. Kalivas P. W. (1999). Glutamatergic and dopaminergic afferents to the prefrontal cortex regulate spatial working memory in rats. Neuroscience 92, 97–106. 10.1016/S0306-4522(98)00747-7 10392833

[B74] Rudd K. E. Johnson S. C. Agesa K. M. Shackelford K. A. Tsoi D. Kievlan D. R. (2020). Global, regional, and national sepsis incidence and mortality, 1990–2017: Analysis for the global burden of disease study. Lancet 395, 200–211. 10.1016/S0140-6736(19)32989-7 31954465 PMC6970225

[B75] Sankowski R. Mader S. Valdés-Ferrer S. I. (2015). Systemic inflammation and the brain: Novel roles of genetic, molecular, and environmental cues as drivers of neurodegeneration. Front. Cell. Neurosci. 9, 28–20. 10.3389/fncel.2015.00028 25698933 PMC4313590

[B76] Savio L. E. B. Andrade M. G. J. de Andrade Mello P. Santana P. T. Moreira-Souza A. C. A. Kolling J. (2017). P2X7 receptor signaling contributes to sepsis-associated brain dysfunction. Mol. Neurobiol. 54, 6459–6470. 10.1007/s12035-016-0168-9 27730511

[B77] Savio L. E. B. Mello P. de A. da Silva C. G. Coutinho-Silva R. (2018). The P2X7 receptor in inflammatory diseases: Angel or demon? Front. Pharmacol. 9, 52. 10.3389/fphar.2018.00052 29467654 PMC5808178

[B78] Schwalm M. T. Pasquali M. Miguel S. P. Dos Santos J. P. A. Vuolo F. Comim C. M. (2014). Acute brain inflammation and oxidative damage are related to long-term cognitive deficits and markers of neurodegeneration in sepsis-survivor rats. Mol. Neurobiol. 49, 380–385. 10.1007/s12035-013-8526-3 23990375

[B79] Semmler A. Frisch C. Debeir T. Ramanathan M. Okulla T. Klockgether T. (2007). Long-term cognitive impairment, neuronal loss and reduced cortical cholinergic innervation after recovery from sepsis in a rodent model. Exp. Neurol. 204, 733–740. 10.1016/j.expneurol.2007.01.003 17306796

[B80] Shieh C. H. Heinrich A. Serchov T. van Calker D. Biber K. (2014). P2X7-dependent, but differentially regulated release of IL-6, CCL2, and TNF-α in cultured mouse microglia. Glia 62, 592–607. 10.1002/glia.22628 24470356

[B81] Shimizu I. Adachi N. Liu K. Lei B. Nagaro T. Arai T. (1999). Sepsis facilitates brain serotonin activity and impairs learning ability in rats. Brain Res. 830, 94–100. 10.1016/S0006-8993(99)01396-7 10350563

[B82] Singer M. Deutschman C. S. Seymour C. Shankar-Hari M. Annane D. Bauer M. (2016). The third international consensus definitions for sepsis and septic shock (sepsis-3). JAMA - J. Am. Med. Assoc. 315, 801–810. 10.1001/jama.2016.0287 PMC4968574 26903338

[B83] Soreq H. Seidman S. (2001). Acetylcholinesterase — New roles for an old actor. Nat. Rev. Neurosci. 2, 294–302. 10.1038/35067589 11283752

[B84] Stanley E. M. Wilson M. A. Fadel J. R. (2012). Hippocampal neurotransmitter efflux during one-trial novel object recognition in rats. Neurosci. Lett. 511, 38–42. 10.1016/j.neulet.2012.01.033 22306091 PMC3288804

[B85] Tuon L. Comim C. M. Petronilho F. Barichello T. Izquierdo I. Quevedo J. (2008). Time-dependent behavioral recovery after sepsis in rats. Intensive Care Med. 34, 1724–1731. 10.1007/s00134-008-1129-1 18542919

[B86] van Gool W. A. van de Beek D. Eikelenboom P. (2010). Systemic infection and delirium: When cytokines and acetylcholine collide. Lancet 375, 773–775. 10.1016/S0140-6736(09)61158-2 20189029

[B87] Virgilio F. D. Vultaggio-poma V. Falzoni S. Giuliani A. L. (2023). Extracellular ATP: A powerful inflammatory mediator in the central nervous system. Neuropharmacology 224, 109333. 10.1016/j.neuropharm.2022.109333 36400278

[B88] Von Bernhardi R. Ramírez G. De Ferrari G. V. Inestrosa N. C. (2003). Acetylcholinesterase induces the expression of the β-amyloid precursor protein in glia and activates glial cells in culture. Neurobiol. Dis. 14, 447–457. 10.1016/j.nbd.2003.08.014 14678761

[B89] Wang H. Hong L. J. Huang J. Y. Jiang Q. Tao R. R. Tan C. (2015). P2RX 7 sensitizes Mac-1/ICAM-1-dependent leukocyte-endothelial adhesion and promotes neurovascular injury during septic encephalopathy. Cell Res. 25, 674–690. 10.1038/cr.2015.61 25998681 PMC4456628

[B90] Wang K. Sun M. Juan Z. Zhang J. Sun Y. Wang G. (2022). The improvement of sepsis-associated encephalopathy by P2X7R inhibitor through inhibiting the omi/HtrA2 apoptotic signaling pathway. Behav. Neurol. 2022, 3777351. 10.1155/2022/3777351 35126784 PMC8813303

[B91] Widmann C. N. Heneka M. T. (2014). Long-term cerebral consequences of sepsis. Lancet Neurol. 13, 630–636. 10.1016/S1474-4422(14)70017-1 24849863

[B92] Yu Y. Liu L. Xie K. Chen H. Dong X. Li Y. (2014). Inhalation of hydrogen gas attenuates brain injury in mice with cecal ligation and puncture via inhibiting neuroinflammation, oxidative stress and neuronal apoptosis. Brain Res. 1589, 78–92. 10.1016/j.brainres.2014.09.030 25251596

[B93] Zaghloul N. Addorisio M. E. Silverman H. A. Patel H. L. Valdés-Ferrer S. I. Ayasolla K. R. (2017). Forebrain cholinergic dysfunction and systemic and brain inflammation in murine sepsis survivors. Front. Immunol. 8, 1673. 10.3389/fimmu.2017.01673 29326685 PMC5736570

