# Supplementary material for: P2X7 receptor contributes to long-term neuroinflammation and cognitive impairment in sepsis-surviving mice
Source: Front Pharmacol. 2023 Apr 21;14:1179723. doi: 10.3389/fphar.2023.1179723 (PMC10160626; doi:10.3389/fphar.2023.1179723)
Supplement: Supplementary file 1 [file Table1.DOCX]

Supplementary Material

**P2X7 receptor contributes to long-term neuroinflammation and cognitive impairment in sepsis-surviving mice**

**Vinícius Santos Alves^1^, Joyce Pereira da Silva^1^, Fabiana Cristina Rodrigues^1^, Suzana Maria Bernardino Araújo^2^, André Luiz Gouvêa de Souza^1^, Raíssa Leite-Aguiar^1^, Stephanie Alexia Cristina Silva Santos^1^, Milla Souza Pessoa da Silva^1^, Fernanda Silva Ferreira^3^, Eduardo Peil Marques^3^, Beatriz Amanda Barbosa Rangel dos Passos^4^, Tatiana Maron-Gutierrez^4^, Eleonora Kurtenbach^1^, Robson da Costa^2^, Simon C. Robson^4^, Claúdia Pinto Figueiredo^2^, Angela T.S. Wyse^3^, Robson Coutinho-Silva^1^, Luiz Eduardo Baggio Savio^1,^***

^1^Instituto de Biofísica Carlos Chagas Filho, Universidade Federal do Rio de Janeiro, Rio de Janeiro, Brazil.

^2^Faculdade de Farmácia, Universidade Federal do Rio de Janeiro, Rio de Janeiro, Brazil

^3^Laboratório de Neuroproteção e Doenças Metabólicas, Departamento de Bioquímica, ICBS, Universidade Federal do Rio Grande do Sul, Porto Alegre, RS, Brazil.

^4^Laboratório de Imunofarmacologia, Instituto Oswaldo Cruz, Fiocruz, Rio de Janeiro, Brazil

^5^Department of Anesthesia, Beth Israel Deaconess Medical Center, Harvard Medical School, Harvard University, Boston, MA, USA

***Correspondence:**Luiz Eduardo Baggio Savio, Ph.D.

E-mail: savio@biof.ufrj.br

Address: Instituto de Biofísica Carlos Chagas Filho – Universidade Federal do Rio de Janeiro, Edifício do Centro de Ciências da Saúde, Bloco G. Av. Carlos Chagas Filho, 373. Cidade Universitária, Ilha do Fundão, Rio de Janeiro, RJ, 21941-902 – Brazil.

Phone: + 55 21 3938 6565; Fax: +55 21 2280 8193.

ORCID ID: <https://orcid.org/0000-0002-6712-6885>

## Supplementary Figures


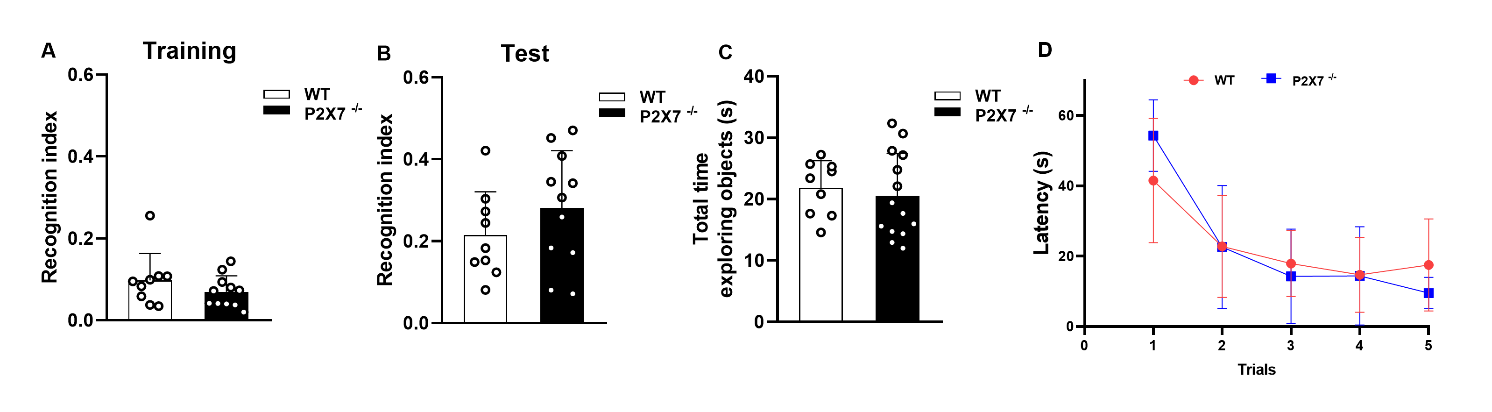


**Supplementary Figure 1. WT and P2X7^-/-^ naive mice did not demonstrate basal difference in NORT and WTMT.** WT and P2X7^-/-^ naïve mice were subjected to the novel object recognition test (NORT) to analyze their memory index. Training with familiar objects (A), test with familiar and novel objects (B), and total time exploring objects(C). WT and P2X7^-/-^ naïve mice were subjected to the Water T-maze test (WTMT) to analyze their spatial working memory(D). Data are expressed as mean ± SEM; One-way ANOVA followed by Tukey's post hoc for recognition index and Two-way ANOVA followed by Tukey's post hoc for WTMT. (n=7-10). The recognition index was calculated by subtracting the time exploring the familiar object from the time exploring the novel object, divided by the total exploration time.
